# Screening of Cognitive Impairment in Patients with Multiple Sclerosis: A Cross-Sectional Study in Georgia

**DOI:** 10.1155/2021/5591078

**Published:** 2021-05-27

**Authors:** Nazibrola Botchorishvili, Nino Shiukashvili, Nina Mikeladze, Ann Dzagnidze, Nino Mikava, Maia Tighashvili, Marina Janelidze

**Affiliations:** ^1^Tbilisi State Medical University, Vazha Pshavela Avenue 33, Tbilisi 0177, Georgia; ^2^School of Natural Sciences and Medicine, Ilia State University, Kakutsa Cholokashvili Avenue 3/5, Tbilisi 0162, Georgia; ^3^S. Khechinashvili University Hospital, Chavchavadze Avenue 33, Tbilisi 0179, Georgia

## Abstract

Cognitive impairment (CI) is a common symptom of multiple sclerosis (MS), with a significant negative impact on the occupational and social functioning of patients. This study aimed to estimate the prevalence and characteristics of CI among MS patients in Georgia. Sixty-eight patients with MS attending a neurology outpatient clinic in Tbilisi, Georgia, were enrolled in the study. Cognitive status was evaluated using two screening tools: the Brief International Cognitive Assessment for MS and the Montreal Cognitive Assessment. The overall prevalence of CI in our MS patients was 47%. We found negative associations between cognitive test results and patients' age, disability status, and depression. Lower education, higher scores on the Expanded Disability Status Scale, and the progressive course of MS were the main predictors of CI in the logistic regression analysis. This is the first study in Georgia to evaluate CI in patients with MS. The prevalence of CI in our study was comparable with those reported in other countries; however, we found greater impairment of the executive system compared to other cognitive domains. In our study, patients who were on continuous DMT showed significantly better performance on the cognitive tests used, indicating possible favorable effect of immunomodulatory drugs on cognition.

## 1. Introduction

Multiple sclerosis (MS) is a chronic demyelinating and degenerative disease of the central nervous system [1]. According to estimates from the Global Burden of Disease Study, there were over two million MS patients in 2016 [2]. MS most commonly affects young and middle-aged adults [3], and women are two to three times more likely to be diagnosed with MS than men [4].

The clinical presentation of MS is diverse, depending on the number and location of demyelinated lesions as well as the extent of gray matter atrophy [5]. The first clinical presentation of MS is referred to as clinically isolated syndrome (CIS). The majority of patients develop relapsing-remitting multiple sclerosis (RRMS), characterized by alternating episodes of relapse and remission. Most RRMS patients eventually transition to a secondary progressive multiple sclerosis (SPMS), characterized by progressive worsening of neurologic functions. Approximately 10% of patients develop primary progressive multiple sclerosis (PPMS). PPMS is identified by steadily worsening neurologic functions from the onset of the disease.

Cognitive impairment (CI) is one of the common features of MS. CI prevalence rates vary markedly across studies, ranging between 22% and 70% [6–8]. From 12% to 57% of patients with clinically isolated syndromes have some degree of cognitive deficit [9], and several studies have demonstrated the presence of CI at the preclinical stage [10–12], although it is more prevalent in patients with progressive MS. In a large multicenter study by Ruano et al., the prevalence of CI in patients with secondary and primary progressive MS was 79.4% and 91.3%, respectively [7]. Planche et al. found that patients with progressive MS have more frequent and severe impairment of information processing speed, executive function, verbal episodic memory, visuospatial abilities, verbal fluency, and working memory, compared to patients with RRMS [13].

The severity of CI in MS varies considerably. The majority of patients develop mild to moderate cognitive decline. MS-related dementia has been reported in 10% to 15% of cases [14], and longitudinal studies have demonstrated progression of cognitive dysfunction over time [15]. Known risk factors for cognitive decline are male sex, younger age at the time of diagnosis, lower education level, and smoking [16]. The severity of CI is associated with the volume of T2-hyperintense and T1-hypointense brain lesions as well as with cortical and deep gray matter atrophy [17]. The most commonly reported cognitive problems are related to information processing speed, episodic memory, visuospatial perception, verbal fluency, and executive function. By contrast, intelligence and basic verbal skills such as expression and comprehension are generally unaffected [18].

CI negatively impacts the occupational and social functioning of MS patients [19]. It has been estimated that up to 80% of MS patients become unemployed at some point, and the majority (70%–80%) retire within five years of diagnosis [20]. Despite its high prevalence, CI often remains overlooked by neurologists. Among the reasons is the distinctive nature of cognitive dysfunction in MS, for which the diagnosis requires the application of specific tests [21].

Evidence suggests that disease-modifying treatment (DMT) has a favorable effect on CI in patients with MS [22]. Studies on cognitive rehabilitation programs that specifically address distinct cognitive domains, such as working memory, attention, or information processing speed, have also reported positive results [23]. Timely identification and management of CI can prevent cognitive deterioration and improve quality of life in patients with MS.

Georgia is a small country, with a population of 3.7 million, located at the crossroads of western Asia and Eastern Europe. It is bounded by Black Sea, Russia, Turkey, Armenia, and Azerbaijan. The prevalence of MS in Georgia is unknown since no epidemiological studies are available. According to experts' statements, the presumed number of MS patients in our country does not exceed 1200 [24]. Standardized neuropsychological assessment is not implemented in routine clinical practice. We have no data on the burden of cognitive dysfunction in Georgian patients with MS.

The objective of our study was to evaluate the prevalence and features of cognitive impairment in Georgian patients with multiple sclerosis.

## 2. Materials and Methods

### 2.1. Subjects

68 MS patients and 68 healthy controls (HCs) were included in the study. All MS patients admitted to the neurology outpatient clinic at S. Khechinashvili University Hospital in Tbilisi, Georgia, from March 1, 2019, to November 1, 2020, were requested to participate in the study. Of 81 patients contacted, 68 (84%) accepted. The following inclusion criteria were applied: (1) willingness and ability to give informed consent; (2) a confirmed diagnosis of MS according to the McDonald criteria (2017 revision); (3) age ≥18 years; (4) no evidence of relapse for at least one month preceding the evaluation; (5) no history of other medical conditions known to affect cognitive abilities; and (6) proficiency in the Georgian language.

An age-, sex-, and education-matched controls were subselected from a large group of 178 individuals who participated in the BICAMS validation study. The following inclusion criteria were applied: (1) age ≥18 years; (2) no history of neurological and psychiatric disease or severe head trauma; and (3) proficiency in the Georgian language.

All participants signed an informed consent form. The study protocol and the informed consent form were approved by the Ethics Committees of S. Khechinashvili University Hospital and Tbilisi State Medical University.

### 2.2. Assessment

We collected patient demographic data, including age, sex, education, and employment status, as well as current treatment, relapse rate, duration, and subtype of the disease from medical records. All subjects underwent standardized neurological examination to define their Expanded Disability Status Scale (EDSS) score. Cognitive status was assessed with two instruments that have been validated in the Georgian language [25]. The Brief International Cognitive Assessment for MS (BICAMS) was introduced by an international expert committee as an effective tool for assessing and monitoring cognitive functions in patients with MS [24, 26]. The battery consists of three tests: the Symbol Digit Modality Test (SDMT), which evaluates information processing speed, the first five trials of the California Verbal Learning Test, second edition (CVLT-II), which assesses verbal memory, and the first three trials of the Brief Visuospatial Memory Test-Revised (BVMT-R), which examines visuospatial memory. All three tests can be administered in 15 minutes and are appropriate for use by neurologists and other healthcare professionals [26].

The Montreal Cognitive Assessment (MoCA) is a well-recognized screening tool for mild cognitive impairment [27]. The test evaluates cognitive domains commonly affected in MS patients, such as executive function, visuospatial ability, attention/concentration, verbal fluency, and memory. It has demonstrated efficacy in evaluating cognitive dysfunction accompanying several chronic neurological disorders, including MS [28, 29]. Finally, the patients' mental health was evaluated using the Beck Depression Inventory (BDI) [30].

All assessments were conducted on the same day and by the same neurologist. After the neurological examination patients were administered the BICAMS battery in the recommended sequence: SDMT, BVMT-R, and CVLT-II, followed by MoCA and BDI. All assessments were applied in succession without interval. All 68 patients completed the SDMT and CVLT-II. Two patients were unable to complete BVMT-R due to motor deficiency in the dominant hand. Fifty-seven patients (84%) completed the MoCA, while the BDI was completed by 55 patients (81%). The reason for missing data was patients' refusal to continue the assessment either due to shortage of time or exhaustion.

Patients were classified as cognitively impaired if their score on any BICAMS test or MoCA was below 1.5 SD of the mean score of the control group. A BDI score ≥19 points was used to define moderate-to-severe depression.

Two MoCA subtests, namely, trial making and abstract thinking, evaluate executive system functioning. Patients with scores of zero on either subtest were considered to have executive dysfunction. We also considered scores of zero to indicate failure on the verbal fluency subtest.

### 2.3. Statistical Analysis

All analyses were performed using SPSS software, version 26.0. Statistical significance was set at *p* < 0.05. Education was tested as both a continuous variable and in categorical format, comparing 15 or more years (higher education) versus general or vocational education. Numerical variables were presented as the mean and standard deviation, and Pearson's correlation coefficient was used to assess correlations. Categorical variables were presented as percentages, and comparisons between CI groups were performed using the chi-square test. *T*-test was used to compare mean scores between groups. Logistic regression analysis was performed to determine the independent predictors of CI. Age, education, disease duration, relapse rate, progressive course, DMT, and EDSS score were used as covariates.

## 3. Results

### 3.1. Patient Demographics and Clinical Data

Demographic and clinical characteristics of the study population are illustrated in Table 1. Overall, patients were predominantly female (71%) and had a mean age of 39.2 ± 9.9 years. RRMS was the most common MS subtype (76%), with only four patients (6%) diagnosed with PPMS. The mean duration for all MS subtypes was 7.0 ± 5.7 years, with 53 patients (78%) having a disease duration of ≤10 years and seven (10%) having less than a one-year history of MS.

Forty-one patients (60%) had never received disease-modifying therapy (DMT). Among the 27 treated patients, sixteen (59%) received S1P receptor agonist, 6 patients (22%) were treated with an anti-CD20 monoclonal antibody, and 5 patients (19%) were on beta-interferons or glatiramer acetate. Half of them (48%) have been treated continuously after the onset of disease and another half received disease-modifying drugs for at least the last five years.

Moderate-to-severe depression was identified in fourteen patients (25.5%). None of the patients were taking antidepressants or other psychotropic medications.

### 3.2. Results of Cognitive Assessments

MS patients demonstrated significantly lower scores on all cognitive tests (Table 2).

The overall prevalence of CI in our sample MS patients was 47% (32 patients). Twenty-nine patients (43%) had abnormal scores on at least one BICAMS test. Abnormal CVLT-II scores were reported for 22 patients (32%), 19 patients (28%) had low SDMT scores, and 15 patients (23%) had poor performance on the BVMT-R. Thirteen patients (23%) received below the cutoff score on the MoCA and were considered cognitively impaired. Only three subjects with normal scores on the BICAMS subtests were classified as cognitively impaired by their MoCA results. Overall, 39% of patients failed at least one of the MoCA executive subtests, making executive function the most commonly affected cognitive domain in our cohort. 18% of patients had impaired verbal fluency.

**Figure 1:**
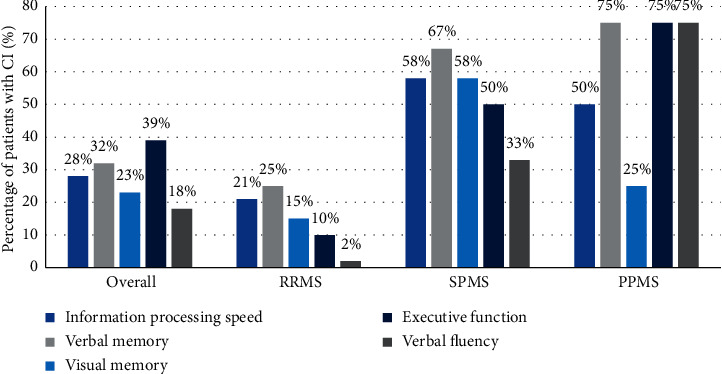
Cognitive impairment in patients with multiple sclerosis.

The highest prevalence of CI was identified in patients with progressive MS, 67% of SPMS patients and 75% of PPMS patients were regarded as cognitive impaired. In the RRMS subgroup, CI was diagnosed in 21 of the 52 patients (40%). Patients with progressive MS demonstrated a higher prevalence of executive dysfunction (Figure 1).

The prevalence of CI was higher in patients with lower education levels (78%) compared with those having higher educational levels (36%) and in those with clinically significant depression (75%). Unemployment was higher in patients with CI than in those without, with a prevalence of 50% and 24%, respectively.  Table 3 demonstrates general characteristics of cognitively impaired and cognitively intact MS patients.

Treatment-naïve MS patients performed worse on all cognitive tests except for the BVMT-R (Table 4). Among these patients, 21 (51%) were cognitively impaired and had a higher prevalence of depression (27% vs. 11%) (Table 5). However, the chi-square test of independence did not show a significant association between CI and treatment status (*X*^*2*^ = 0.71; *p* > 0.05).

We found a significant negative correlation between all cognitive tests and EDSS (Table 6). SDMT was the only test that was negatively correlated with age and BDI scores. All cognitive tests except CVLT-II were positively correlated with education. We did not find a significant correlation between CI and disease duration, although the prevalence of CI was somewhat higher (50% vs. 46%) in patients with longer (≥11) disease duration.

We used logistic regression analysis to identify predictors of cognitive impairment and found a significant association between lower education, higher EDSS score, progressive disease course, and cognitive decline in MS patients (Table 7).

## 4. Discussion

The reported prevalence of CI in MS patients varies between 22% and 70% and is affected by factors, such as patient population, study design, and, particularly, the specific neurocognitive tests used [6–8]. In studies using the BICAMS battery to assess cognitive status, the range of CI is much narrower, from 45% to 67% [31]. In our study, the BICAMS battery by itself identified CI in 43% of MS patients. This is slightly lower but still in the range of data reported in the literature. However, three of our patients with normal results on the BICAMS battery failed the MoCA test, raising the total number of cognitively impaired patients by 10%. Together, the two screening instruments identified CI in 47% of our study population.

The high reliability of the MoCA in MS patients has been demonstrated by several studies. The test has shown compatible psychometric properties with conventional, more extensive neuropsychological tests used in MS patients—the Brief Repeatable Battery (BRB) and the Minimal Assessment of Cognitive Function in Multiple Sclerosis (MACFIMS) [28, 29]. We found a good correlation between BICAMS and MoCA results and feel that the simultaneous application of these instruments increases their diagnostic capacity. To the best of our knowledge, there is only one study that has reported the use of MoCA to estimate the prevalence of CI in MS patients. In that study, Gómez-Moreno et al. [32] evaluated 52 patients with MS using MoCA and BRB-N, identifying CI in 25% and 21% of their sample, respectively.

CI in MS shows specific phenotypes, mainly affecting domains, such as information processing speed, memory, attention, and executive function. Information processing speed is considered to be the most commonly impaired cognitive sphere in MS populations, being present in 40%–70% of patients [18]. In contrast, only 28% of our study participants had impairment of information processing speed as assessed by the SDMT test, making it the third most affected domain after executive function and verbal memory. However, 23 (34%) patients from our cohort were familiar with the SDMT. Among them, the number of participants, who were impaired based on the SDMT, was almost twice as low as those who had never completed the test before. This could explain why our study revealed considerably less extensive impairment of information processing speed.

As mentioned earlier, the most commonly affected cognitive domain among our MS patients was the executive system. One-third of patients (30%) failed on the Trail Making subtest, and 18% had impaired abstraction thinking. Verbal memory was the second commonly affected cognitive sphere, presenting in 32% of subjects, while 23% of patients had impaired visuospatial memory. Verbal fluency was less commonly affected cognitive domain, observed only in 18% of patients.

According to early research, executive dysfunction typically occurs in 15%–25% of patients with MS [33, 34]. However, Drew et al. [35] evaluated 98 MS patients using the Delis–Kaplan Executive Function System (D-KEFS) standardized test set and found some degree of executive system dysfunction in 66% of the study population. Our results are also comparable with those of an Italian multicenter study, involving 1,040 patients with MS, in which executive dysfunction was the second most affected cognitive function after information processing speed, being present in 41% of patients [7]. In that study, the Stroop test was used for the assessment of executive system function. The MoCA was not designed for a thorough evaluation of executive system functioning, but has shown good construct validity with many comprehensive neuropsychological instruments, including D-KEFS, and has been recommended as a reliable screening test for executive dysfunction [27].

CI can develop in patients with any type of MS, even though it is more frequent in progressive forms of the disease [9]. As expected, in our sample, CI and particularly executive dysfunction was more prevalent in patients with progressive MS.

By reducing the inflammation and burden of T1 and T2 brain lesions, disease-modifying therapies may have beneficial effects on cognition [36]. Several studies have demonstrated protective effects of interferons, glatiramer acetate, fingolimod, and natalizumab on cognitive status [37]. In our sample, CI was more prevalent and pronounced in treatment-naïve patients, despite the predominance of patients with higher education in this subgroup. On the other hand, the percentage of patients with clinically significant depression was higher in the treatment-naïve group, which may have influenced the overall cognitive performance.

According to current research, CI in MS has various patient- and disease-related risk factors, including genetic factors, advanced age, male sex, lower cognitive reserves, depression, fatigue, disease duration, younger age at disease onset, and greater brain atrophy [16]. We found that CI was more prevalent in male patients who are not under treatment with immunomodulatory drugs and in those with clinically significant depression. In our study, lower education level, progressive disease course, and higher physical disability were major predictors of cognitive dysfunction.

Connections between disease duration and the risk of CI are still being researched. While longitudinal studies have shown an increasing prevalence of CI over time [15], majority of them have not found associations between CI and disease duration [9, 38]. We did not find a correlation between disease duration and cognitive dysfunction. This observation may indicate that CI is more closely related to individual factors, such as MS subtype and disease severity, than to a cumulative effect over time.

One of the main limitations of our study is the small sample size and underrepresentation of patients with progressive MS. In addition, we did not obtain the results of the MoCA test of 11 patients, and thus, we may have underestimated the prevalence of CI.

## 5. Conclusions

CI appears to be quite prevalent in Georgian patients with MS. We found that patients with progressive MS, higher disability status, or lower levels of education have a higher risk of CI, and their conditions and the possible need for medications should be closely monitored. We found that CI is more frequent in patients with higher BDI scores, indicating a need for mental health monitoring and services. Adequate management of mood disorders may alleviate cognitive symptoms. In our study, patients who were on continuous DMT showed significantly better performance on the cognitive tests used, indicating possible favorable effects of immunomodulatory drugs on cognition. Considering the high prevalence of executive dysfunction in our patient population, additional testing, specifically designed to assess executive system functioning, should be considered in the cognitive assessment of MS patients.

## Figures and Tables

**Table 1:** Characteristics of the study population.

	Patients	Controls
Number of subjects, *n*	68	68
Age (years), mean ± SD	39.2 (±9.9)	38.5 (±9.9)
Female, *n* (%)	48 (71%)	46 (68%)
Male, *n* (%)	20 (29%)	22 (32%)
Education (years), mean ± SD	14.3 ± 2.1	14.5 ± 1.9
Higher education (≥15 years)	50 (74%)	49 (72%)
Lower education (≤14 years)	18 (26%)	19 (28%)
Employed	39 (57%)	57 (84%)
Unemployed	29 (43%)	11 (16%)
Disease duration (years), mean ± SD	7.0 ± 5.7 years	—
EDSS score, mean ± SD	3.3 ± 1.6	—

*MS subtype*		
RRMS, *n* (%)	52 (76%)	—
SPMS, *n* (%)	12 (18%)	—
PPMS, *n* (%)	4 (6%)	—

EDSS: Expanded Disability Status Scale; PPMS: primary progressive multiple sclerosis; RRMS: relapsing-remitting multiple sclerosis; SD: standard deviation; SPMS: secondary progressive multiple sclerosis.

**Table 2:** Comparison of mean scores between patients and healthy controls.

	MS mean ± SD	HC mean ± SD	*T*-score	*p* value
SDMT	35.5 ± 12.7	46.0 ± 11.8	−4.990	<0.0001
CVLT-II	51.0 ± 11.8	58.5 ± 8.2	−4.401	<0.0001
BVMT-R	22.0 ± 8.0	25.6 ± 6.8	−3.011	0.0015
MoCA	22.6 ± 4.0	26.4 ± 4.0	−4.684	<0.0001

BVMT-R: Brief Visual Memory Test-Revised; CVLT-II: California Verbal Learning Test, second edition; HC: healthy control; MoCA: Montreal Cognitive Assessment; MS: multiple sclerosis; SD: standard deviation; SDMT: Symbol Digit Modality Test.

**Table 3:** Characteristics of cognitively impaired and cognitively intact MS patients.

	Cognitively impaired, *N* = 32	Cognitively intact, *N* = 36
Age (years), mean ± SD	41.2 ± 8.9	37.4 ± 10.7
Male	12 (37.5%)	8 (22.2%)
Female	20 (62.5%)	28 (77.8%)
Education <15 years	14 (43.7%)	4 (11.1%)
Education ≥15 years	18 (56.3%)	32 (88.9%)
Unemployed	16 (50%)	23 (64%)
Employed	16 (50%)	13 (24%)
RRMS	21 (65.6%)	31 (86.1%)
SPMS	8 (25%)	4 (11.1%)
PPMS	3 (9.4%)	1 (2.8%)
EDSS, mean ± SD	3.7 ± 1.8	2.8 ± 1.4
Duration (years), mean ± SD	7.3 ± 6.2	6.7 ± 5.1
BDI ≥ 19 points	9 (33.3%)	3 (8.3%)
DMT naïve	21 (66%)	20 (55.6%)
Receiving DMT	11 (34%)	16 (44.4%)

BDI: Beck Depression Inventory; DMT: disease-modifying therapy; EDSS: Expanded Disability Status Scale; MS: multiple sclerosis; PPMS: primary progressive multiple sclerosis; RRMS: relapsing-remitting multiple sclerosis; SD: standard deviation; SPMS: secondary progressive multiple sclerosis.

**Table 4:** Comparison of cognitive test results between MS subtype groups.

	MS patients on DMT mean ± SD	MS patients without DMT mean ± SD	*T*-score	*p* value
SDMT	41.3 ± 12.6	31.7 ± 11.3	−3.3	<0.001
CVLT-II	54.0 ± 12.1	48.9 ± 9.9	−1.9	<0.05
BVMT-R	21.9 ± 7.6	21.7 ± 8.3	−0.1	0.4
MoCA	23.4 ± 3.0	21.9 ± 4.6	−1.3	0.09

BICAMS: Brief International Cognitive Assessment for MS; BVMT-R: Brief Visual Memory Test-Revised; CVLT-II: California Verbal Learning Test, second edition; MoCA: Montreal Cognitive Assessment; SD: standard deviation; SDMT: Symbol Digit Modality Test.

**Table 5:** Characteristics of MS patients with and without DMT.

	MS patients on DMT	MS patients without DMT
Number of subjects, *n* (%)	27 (40%)	41 (60%)
Age (years), mean ± SD	37.6 ± 9.2	40.3 ± 10.4
Female, *n* (%)	18 (66.7%)	30 (73%)
Male, *n* (%)	9 (33.3%)	11 (27%)
Education < 15 years	19 (70%)	20 (49%)
Education ≥15 years	8 (30%)	21 (51%)
Unemployed	12 (44%)	20 (49%)
Employed	25 (55%)	35 (59%)

*MS subtype*		
RRMS	23 (85%)	29 (70.7%)
SPMS	4 (15%)	8 (19.5%)
PPMS	—	4 (9.8%)

Disease duration (years), mean ± SD	6.7 ± 4.6	7.1 ± 6.5
EDSS (years), mean ± SD	2.6 ± 1.3	3.7 ± 1.7
BDI ≥ 19	3 (11%)	11 (27%)
CI	11 (41%)	21 (51%)

BDI: Beck Depression Inventory; CI: cognitive impairment; DMT: disease-modifying therapy; EDSS: Expanded Disability Status Scale; PPMS: primary progressive multiple sclerosis; RRMS: relapsing-remitting multiple sclerosis; SD: standard deviation; SPMS: secondary progressive multiple sclerosis.

**Table 6:** Correlation of cognitive tests with patient characteristics and EDSS and BDI scores.

	Age		Education		Duration		EDSS		BDI	
	*r*	*p* value	*r*	*p* value	*r*	*p* value	*r*	*p* value	*r*	*p* value
SDMT	−0.40	<0.001	0.24	0.04	−0.17	0.1	−0.58	<0.001	−0.28	0.032
CVLT	−0.11	0.4	0.20	0.09	−0.10	0.4	−0.40	<0.001	−0.15	0.7
BVMT	−0.21	0.07	0.29	0.01	−0.12	0.3	−0.34	<0.001	−0.06	0.7
MoCA	−0.19	0.2	0.25	0.04	−0.13	0.3	−0.44	<0.001	−0.24	0.7

BDI: Beck Depression Inventory; BVMT-R: Brief Visual Memory Test-Revised; CVLT-II: California Verbal Learning Test, second edition; EDSS: Expanded Disability Status Scale; MoCA: Montreal Cognitive Assessment; *r*: Pearson correlation coefficient; SDMT: Symbol Digit Modality Test.

**Table 7:** Logistic regression analysis for various factors predicting CI in patients with MS.

	Coefficient	Odds ratio	95% CI	*p*
Age	0.0390	1.04	0.9892, 1.0929	0.1256
Lower education	1.8281	6.22	1.7786, 21.7673	0.0042
Duration	0.0178	1.01	0.9348, 1.1085	0.6825
EDSS	0.3880	3.24	1.0544, 2.0606	0.0232
Progressive course	1.1779	3.24	0.9846, 10.7122	0.0531
Relapse rate	0.4613	0.6305	0.3481, 1.1419	0.1280

DMT: disease-modifying therapy; CI: confidence interval; EDSS: Expanded Disability Status Scale.

## Data Availability

The data used to support the findings of this study are available from the corresponding author (NB) upon request.
